# A 16 Month Survey of Cyclosporine Utilization Evaluation in Allogeneic Hematopoietic Stem Cell Transplant Recipients

**Published:** 2016

**Authors:** Maria Tavakoli Ardakani, Ali Tafazoli, Mahshid Mehdizadeh, Abbas Hajifathali, Simin Dadashzadeh

**Affiliations:** a *Department of Clinical Pharmacy, School of Pharmacy and Pharmaceutical Sciences Research Center, Shahid Beheshti University of Medical Sciences, Tehran, Iran. *; b *Students’ Research Committee, School of Pharmacy, Shahid Beheshti University of Medical Sciences, Tehran, Iran.*; c *Department of Bone Marrow Transplant, Taleghani Hospital, Shahid Beheshti University of Medical Sciences, Tehran, Iran. *; d *Department of Pharmaceutics, School of Pharmacy and Pharmaceutical Sciences Research Center, Shahid Beheshti University of Medical Sciences, Tehran, Iran.*

**Keywords:** Administration, adverse effects, cyclosporine, drug utilization evaluation, graft versus host disease, hematopoietic stem cell transplantation

## Abstract

**Objectives:**

Graft versus host disease (GVHD) is a life threatening reaction in the stem cell transplantation process. Nowadays Cyclosporine is the most commonly utilized agent for GVHD prophylaxis and it has a major role in successful transplantation. Cyclosporine has been applied for many years in this field but it could be stated that currently no general consensus is available for its optimal method of administration. Conditions related to cyclosporine administration and possible related adverse reactions observed closely in our patients with the aim of constructing a comprehensive practice guideline in the future.

**Patients and Methods:**

Allogeneic stem cell transplant recipients who have been taking cyclosporine were monitored during and after their hospitalization while recording all observations on predefined questionnaires on the basis of periodic clinical and laboratory examinations for a 16 month period.

**Results:**

Mean recorded duration of infusions was 1.44 ± 0.68 h and by twice daily administration, means intravenous and oral dose was 101.85 ± 22.03 mg and 219.28 ± 63.9 mg, respectively. A mean CsA trough level after about 12 h of specified unique doses was 223 ± 65 ng/mL. We found hypertension, nephrotoxicity, neurotoxicity, hypertension, and dyslipidemia in about 14, 20, 48, and 94 percent of patients.

**Conclusions:**

This study proposed that permanent guidance of healthcare team according to a fixed and standard method of cyclosporine administration routine with using efficient facilities and protocols would be helpful considerably for an optimal pharmacotherapy.

## Introduction

Graft-versus-host disease (GVHD) is a common and hazardous complication after allogeneic hematopoietic stem cell transplantation (HSCT) which is associated with high rates of morbidity and mortality.(1, 2) Cyclosporine (CsA)-based immunosuppression is a worldwide acceptable prophylactic and therapeutic regimen in this field. CsA utilization is complicated by its narrow therapeutic index (3) and it is common for practitioners to change the dosage because of observation of toxicities or inadequacy of treatment especially in early post-transplant period. Excessive cyclosporine blood levels may cause significant toxicity and cause temporary cessation or discontinuation of cyclosporine. Low concentrations also are associated with significant immunologic reactions, most importantly graft-versus-host disease and stem cell graft lost. The pharmacokinetic behavior of cyclosporine is also highly variable which makes sustaining a concentration within therapeutic window so challenging.(4) Any obstacle in achieving the blood concentration targets can result in serious immunologic complications such as GVHD or poor engraftment as well as hypertension, hyperglycemia, renal dysfunction, and central nervous system complications or infections (4) therefore close observation and handling of all deviations from standard CsA administration is obviously vital.

Regarding high occurrence of toxicities of CsA and graft related reactions, periodic drug utilization evaluation (DUE) studies to evaluate its rational and proper use considering day-by-day augmenting available literature will be helpful in providing lifesaving practices.

## Experimental

Between April 2013 and July 2014, all patients candidate for allogeneic hematopoietic stem cell transplantation in Taleghani transplantation center, a teaching and referral hospital in Tehran, Iran, were evaluated for enrollment in this study. Inclusion criteria was age lower than 65, stable kidney function (baseline serum creatinine lower than 2 mg/dL), no liver dysfunction (more than 2 times upper limits of normal for transaminases and bilirubin), baseline blood pressure within normal values (systolic blood pressure lower than 120 mmHg or diastolic blood pressure lower than 80 mmHg and not lower than 90/60 mmHg), feasibility of oral intake, taking standard conditioning regimen for allogeneic HSCT, taking standard CsA based immunosuppression for GVHD prophylaxis. Exclusion criteria consisted of mental or psychiatrically uncooperative patients, pregnant or lactating patients, and HIV + ones. All included patients have been observed during their admission and afterwards, at least for 100 days after stem cell infusion (according to acute GVHD definition) without any drop outs and their clinical and laboratory data observed for CsA utilization evaluation. We recoded all patients’ baseline demographic data, age, gender, weight, height, and underlying disease. During in-patient state that took generally more than 3 weeks, vital signs and clinical presentations checked 4 times daily. Some of critical laboratory tests on blood cell counts and biochemistry values evaluated twice daily and the others obtained twice weekly like cyclosporine, albumin and C-reactive protein levels as the routine protocol. Only data that was highly relevant to this study scope extracted and included in the analysis. Patients came for follow up clinic visits at least once weekly or more according to physicians order until the day +100 of HSCT after discharge. The end point of the study was +100 post transplantation day of the last enrolled patient.

Our protocol for GVHD prophylaxis was based on European Group for Blood and Marrow Transplantation (EBMT)-European Leukemia Net (ELN) working group recommendations and Food and Drug Administration (FDA) instructions for CsA administration and standardized practice and National Cancer Institute (NCI) tables generally used for adverse effects grading.(5)

Predefined questionnaires containing patients’ demography, drug history, medical background, and laboratory tests before and after enrollment used for data record. Kidney and liver function test, blood lipid and glucose levels and clinical observation of neurotoxicity and hypertension were monitored meticulously. Clinical events especially occurrence of GVHD or veno-occlusive disease (VOD) were also assessed on a daily basis during the admission and periodically after discharge in our transplantation clinic.

Information related to CsA administration conditions regarding duration of infusion, infusion sets, carrier solutions and acute infusion reactions recorded separately. All acquired data analyzed with SPSS version 17.0 software. The “analyze” tab used to obtain basic descriptive statistics of our data. For scale” data like age, height, weight, dosing and administration parameters and laboratory measurements “descriptive” command implemented for achieving mean, median and standard deviation values. Summarization of “categorical” data including demonstration of percentages for occurrence of adverse effects has been done with “frequencies” command. 

All of our interventions were implemented after approval by “Shahid Beheshti University of Medical Sciences” ethical committee. 

## Results

Descriptive analysis of 35 patients’ demography showed 18 males (51.4%) and 17 females (48.6%) with median ± SD age of 27 ± 8.08 years (range 18 to 50 years) and height of 1.67 ± 0.10 meters and weight of 70.5 ± 14.3 kilograms. There were only two non-Iranian patients. Underlying diseases were acute myeloid leukemia (n = 20, 57.1%), acute lymphoblastic leukemia (n = 11, 31.4%), aplastic anemia (n = two, 5.7%), non-Hodgkin lymphoma (n = one, 2.9%), and adrenoleukodystrophy (n = one, 2.9%). Summary of demographic data could be found in Table 1.

**Table 1:** Summary of demographic data for study population

	**Mean ± SD**
**Age (years)**	29±8.08
**Weight (Kg)**	70.5±14.33
**Height (m)**	1.67±0.10
**Gender**	**Number (Percent)**
Female	17 (51.4%)
Male	18 (48.6%)
**Underlying disease**	
Acute myeloid leukemia	20 (57.1%)
Acute lymphoblastic leukemia	11 (31.4%)
Aplastic anemia	2 (5.7%)
Non-Hodgkin lymphoma	1 (2.9%)
Adrenoleukodystrophy	1 (2.9%)
**Race**	
Iranian	33 (94.2%)
Non-Iranian	2 (5.7%)

Focusing on administration status loading dose was not administered for any patient based on our center protocol. Infusion pump has been used only in 10 (28.6%) of transplanted patients. Mean duration of infusions was 1.44 ± 0.68 (mean ± SD) with a range of 0.5 to three h. All patients took CsA in normal saline solution using hospital grade Lifen® plastic tubing. 25 of patients received their doses exactly 12 h after previous dose on random checking for preciseness of twice daily dosing.

CsA was administered at intravenous doses ranging from 100 mg/day to 250 mg/day with the mean of 101.85 ± 22.03 mg/dose (mean ± SD). Oral CsA was started after patient stabilization and oral tolerability occurred on about14 days post transplantation with a range of 10 to 25 days. Mean dose for every 12 h administration of oral dosage form was 219.28 ± 63.9 mg (mean ± SD). Mistaken over-dosage (more than twice the ordered dose) in a single administration discovered only for one patient.

Laboratory observations showed a mean CsA trough level after about 12 h of specified unique doses of 223 ± 65 ng/mL (mean ± SD). A single occasion of sub-therapeutic level observed in four (11.4 %) and double occasions in five (14.28 %) of patients whereas supra-therapeutic levels found in nine (25.71%), two (5.71%), and one (2.9%) of patients as single, double and triple occasions, respectively. Interestingly two patients had more than five occasions of supra-therapeutic level detection despite frequent dose modifications. Only 15 (about 43%) of patient had constantly within therapeutic range levels during their admission period. Modifications for dosing based on drug levels and other laboratory findings happened for 14 (40%) of cases.

Regarding occurrence of adverse reactions, CsA temporary stop order considered for only one patient due to nephrotoxicity and two patients for intolerable neurotoxicity with general presentation of tremor. Of infusion reactions hot flashes in four, rigors in two, nausea in nine (about 26%), paresthesia in five and headache in two patients had been observed.

Kidney related observations revealed a mean baseline serum creatinine of 0.81 ± 0.06 (mean ± SD) with a range of 0.7 to 0.9 mg/dL. Acute kidney injury occurred in seven patients (three in each of grades one and two and one in grade three) all taking other nephrotoxins. Co-administration of nephrotoxic drugs, specifically, amphotericin and aminoglycosides recorded in 15 and18 patients respectively. No permanent kidney injury due to CsA therapy recorded.

Neurotoxicity classification of patients by NCI toxicity criteria showed 22.9, 5.7, 11.4, and 8.6% of patients in grades one, two, three, and four respectively. 

According to NCI grading, high blood pressure or hypertension defined as an increase by greater than 20 mmHg from baseline or to more than 150/100 mmHg if previously within normal values as mentioned in inclusion criteria. Hypertension after institution of CsA therapy recorded for five patients at least in one occasion, all in grade one NCI grading (asymptomatic, transient increase by greater than 20 mmHg) , except one case in second grade (recurrent increase by greater than 20 mm Hg to > 150/100 mmHg). Only one patient required pharmacologic intervention that was controlled successfully with enalapril.

Of metabolic alterations, dyslipidemia was found in about 94 % of patients consisting of 29 patients with hyper triglyseridemia (triglyseride (TG) > 150 mg/dL) and 24 with hyper cholesterolemia (Low-density lipoprotein- cholesterol (LDL-C) > 100 mg/dL). Therapy for dyslipidemia considered only for four patients. All patients showed hyperglycemia at least in one occasion during admission but only nine (25.7%) received therapy. Treatment was consisted of metformin for three patient and all others received insulin. Using NCI hyperglycemia grading five, 18, 11, and one case presented in grades one, two, three, and four respectively. 

Observations for clinical outcomes showed eight patients (23%) with acute GVHD (two, one, three, and two cases in grades one, two, three, and four respectively) and only one occurrence of VOD during admission. On follow up visits one case of chronic GVHD discovered. All GVHD cases controlled with therapeutic measurements but the VOD case died after a second admission.

Work up for mucositis showed 11, 22, and two patients with mild, moderate and severe mucositis, respectively according to Common Terminology Criteria for Adverse Events (CTCAE) grading.

30 patients returned to clinic for follow up appointments post discharge and included in post-discharge analysis. Clinical and laboratory monitoring was not as close as hospitalization state expectedly. Despite gradual tapering down of oral CsA doses, interestingly higher mean trough levels observed for patients during the first 100 post-transplant days in post-discharge period compared to mean admission trough levels (369 versus 223 ng/mL). As a common finding in our center, observations for some of CsA related toxicities considerably increased after patient discharge. Totally 22 cases (73%) of nephrotoxicity, seven (23%) for neurotoxicity and six (20%) occasions of GI complications including nausea, vomiting and diarrhea observed in follow up clinic visits considering elimination of the conditioning regimen effect by the time passed.

Totally four patients expired due to relapse of underlying disease, veno-occlusive disease and chemotherapy toxicities. Summary of our records could be found in Table 2.

**Table 2:** Summary of in clinical and laboratory observations in the study population

Records	**Number (Percent)**
**Infusion set**	
Gravity filled	25 (71.4%)
Pump	10 (28.6%)
**Infusion reactions**	15 (42.8%)
**Other adverse events **	
Nephrotoxicity	7 (20%)
Neurotoxicity	17 (48.6%)
Hypertension	5 (14.2%)
Hypertriglyceridemia	29 (82.8%)
Hypercholesterolemia	24 (68.5%)
Acute GVHD	8 (22.8%)
VOD	1 (2.6%)
Mucositis	34 (97.1%)
**Dosing**	**Mean ± SD**
IV dose (mg)	101.85 ± 22.03
Oral dose (mg)	219.28 ± 63.9
**Trough concentrations**	**Mean ± SD**
In patients (ng/mL)	223 ± 65
Out patients (ng/mL)	369 ± 234.4

## Discussion

Administration of cyclosporine is the cornerstone of GVHD prophylaxis in allogeneic HSCT. Efficiency of CsA therapy has been demonstrated with significant decrease in GVHD occurrence and improved clinical outcomes. (6) As an eminent organization in HSCT field, European Group for Blood and Marrow Transplantation (EBMT) periodically publishes practice guidelines specifically for GVHD management and we applied their recommendations in our center throughout the study. Also FDA presented prescribing information for Sandimmune® and Neoral® implemented for drug administrations.

Our findings showed that despite the effort for acting in accordance with guidelines, deviations from drug administration protocol were notable. Based on the guideline CsA levels should be kept within 100 and 300 ng/mL therapeutic window especially at early post transplantation phases (first 3-4 weeks), but occasions of outrange drug level values recorded in 19 patients, without any dose modification during our study in seven of them. Common, day to day variations in measurements of CsA blood level, without demonstration of clinical malfunctions and to some extent, unreliability of laboratory tests, could be stated as possible reasons. 

Both intravenous and oral formulation administration tracked for patients. The shortest standard duration of infusion recommended for CsA is two h that endorsed by prescribing information but a major pitfall observed for intravenous CsA administration was excessively faster infusion rates. Some of possible explanations for this issue could be insufficient number of infusion pumps, malfunction of these devices, and application of gravity fill infusion sets instead of pumps which used for many of our cases; Gravity filled sets are potentially exposed to easy manipulability by unobserved curious patients. Considerable variability in administration orders by physicians also could be rooted in variations of protocols among different centers and studies in the literature. 

Faster infusions, regarding the standard recommended time for intravenous cyclosporine administration, may partially explain the high number of cases with infusion reactions in our study. As stated by Gupta *et al*, rate of infusion can influence pharmacokinetics of CsA and consequently be associated to (peak) concentration dependent adverse effects. (7) This fact also confirmed by animal studies. (8)

Possible dose or concentration dependency of CsA adverse effects like nephrotoxicity, neurotoxicity, hypertension (HTN) and metabolic abnormalities has been showed by some studies. (9-11) An interesting finding of our study was that these adverse outcomes were not more frequent in patients with higher mean blood concentrations although limited numbers of our study cases affect a definite judgment. In addition low predictive value of CsA trough concentrations monitoring that applied in our study should be taken into consideration. (12-14)

Close observation of all vital signs and clinical symptoms of patient early after start of administration specifically for the first 30 min recommended by manufacturer label instructions but unfortunately missed for many of our hospitalized patients because of limited number of staff so it could possibly affect patients’ morbidity.

Presence of major intrusive factors besides cyclosporine administration with more significance like occurrence of VOD or GVHD and therapy with hepatotoxic conditioning chemotherapy regimens and other agents like azole antifungals hindered analysis of related liver function lab results. Also stronger etiologies for hyperuricemia which is a well-known adverse effect of cyclosporine existed in our population like hemolytic reactions, possible hemolytic uremic syndrome and frequent transfusions. 

A frequently complication among allogeneic hematopoietic stem cell transplantation patients is hypertension. (15) Increase in blood pressure could happen early after administration of cyclosporine maybe via vascular changes leading to systemic and renal vasoconstriction.(16) Significant number of our study population showed HTN but because of mild and transient nature of alterations, no therapeutic measure used for most of them; It is notable that such findings could also be related to other factors like deliberate hydration for chemotherapies and were not detectable without success in our four times daily blood pressure measurements policy.

An important tormenting syndrome of post-transplant situations is neurotoxicity. 49 and 23% of our patients revealed a range of symptoms of neurotoxicity both during admission and post discharge, respectively. Severe presentation of neurotoxicity including confusion, disorientation, decreased responsiveness, visual hallucinations, delusions, seizures, pyramidal motor weakness, cortical blindness, aphasia and ataxia has been reported in four to 11% HSCT patients (17, 18) and both at therapeutic and toxic CsA levels (17, 19, 20) therefore routine investigation for this adverse effect would be highly valuable.

It should be noted that many of our observations for adverse reactions could be associated with high probability to other co-medications besides cyclosporine like famous busulfan neurotoxicity so records for such events would not show an obligatory cause-and-effect correlation with CsA therapy. Notably suicidal ideation observed in one our patient that put him in the fourth NCI neurotoxicity grade. 

Metabolic complications are troublesome and common adversities of immunosuppressive therapies; namely dyslipidemia can affect long term survival of transplant patients. (21) Cardiovascular risk factors are augmented in allogeneic HSCT patients even more than autologous transplantation, maybe because of differences in immunosuppressive or conditioning regimens especially inclusion of cyclosporine. Generally LDL-C is the major marker of cardiovascular risk, but treatment for severe hypertriglyceridemia also has been recommended. (21) Stratification of LDL-C goals was undertaken in our study according to the National Cholesterol Education Program ATP-III guideline. Statins as the first-line agents for high LDL-C dyslipidemia also proposed for HSCT patients.(21) 94% of study patients demonstrated dyslipidemia but pharmacotherapy implemented only for four of them during hospitalization because our clinicians were reluctant to use therapeutic agents concerning further complicating patients poly pharmacy, imposing new probable adverse effects, and unpredictable alterations in monitoring lab results. Post-discharge lipid profiles were followed periodically and evidence based therapies implemented more freely and deliberately for patients who had the indications. 

HSCT recipients frequently require parenteral nutrition and immunosuppressive drugs like steroids that increase the risk of hyperglycemia as observed in our study population. (22) Glucose metabolism could be affected by cyclosporine via impairment of both synthesis and secretion of insulin, increased insulin clearance, and alterations in insulin sensitivity. (23) Considering the fact that strict glucose control would improve patients’ outcome (24) and the observation of even transient episodes of hyperglycemia in all of our patients, nine of 35 took insulin or metformin which was effectively corrective.

Kidney injury is a common complication and a major concern early after HSCT. (25) Cyclosporine could act as a deteriorating factor for kidney dysfunction, especially when used concomitantly with other nephrotoxic agents like amphotericin B or aminoglycosides. (26) Co-administration of CsA with amphotericin and aminoglycosides (gentamicin or amikacin) recorded in about 43 and 51% of our patients, respectively. Among patients taken such nephrotoxic combinations four of 11 patients with a triple combination had grade two and three toxicity, two patients with similar regimen showed grade one, one of patients taken only AG had grade one but no one with only CsA without AG and amphotericin therapy experienced acute injury. Therefore close monitoring of renal function which is a major concern in our center seems to be a rational and informative approach especially for patients who receive other nephrotoxic agents. 

Several studies can be found in scientific literature that noted unspecifically about cyclosporine utilization evaluation. In a study by Kagawa and colleagues, cyclosporine dosing beside monitoring of blood concentrations and occurrence of clinical events like GVHD or drug adverse effects evaluated. Their study population consisted of only eight allogeneic HSCT recipients with different underlying disease. Patients were followed generally for about 70 to 75 days (50 days after detection of peak cyclosporine blood concentrations with continuous intravenous administration). They found that only one patient had acute GVHD. All patients showed renal dysfunction by measurements of BUN and serum creatinine levels that presented several days after occurrence of maximum CsA blood concentrations. Both of these markers measured and recorded also in our study and we found 20% of nephrotoxicity. Liver function tests altered in two of them at the same situation. No data specifically for post-discharge status and oral administrations presented by these authors. (27). In a four year retrospective study on a cohort of 91 consecutive myeloablative allogeneic HSCT adult patients with focus on correlation of hyperglycemia and clinical outcomes, researchers found about 69% occurrence of hyperglycemia. 49 of these patients had moderate to severe hyperglycemia which was almost superimposable to grade one in our staging system. It could be stated that higher grades of this adverse effect observed more frequently in our study population but considering the transient pattern of our observations, numerous monitoring in our protocol may explain this over detection. (22) In allogeneic HSCT the risk of premature death from cardiac complications predicted to be 2.3-fold higher than general population. Post-transplantation dyslipidemia can have a major role in this issue. Occurrence of dyslipidemia in this transplant estimated to be up to 71%. (21) Despite our data analysis for hypercholesterolemia and hypertriglyceridemia, the major influence of these complications are on long term outcomes of patients therefore design and continuation of such studies will be highly enlightening in HSCT setting. In another study with the aim to explore clinical and radiologic characteristics of cyclosporine related neurotoxicity specifically in HSCT patients, magnetic resonance imaging and computerized tomography scan findings were observed. In our ward the general policy was to maintain environmental isolation for the patients so radiologic evaluations implemented at minimum numbers only for severe cases. Authors found 4.6% occurrence for severe presentations (versus 8.6% in our study) like generalized seizures, occipital blindness and hemiparesis with hyper-intensity lesions in imaging after a prodromal phase including headache and hypertension. (17) Hypertension could be related to several intruding factors in HSCT patients but cyclosporine would always be a major accuser. Number of studies for evaluation of cyclosporine induced hypertension in HSCT setting is so limited. In a study on 112 HSCT, patients randomized to methotrexate or cyclosporine (3 mg/kg/day intravenous that later converted to oral form) GVHD prophylaxis. Loughran and colleagues found a 60% incidence of hypertension in the first 120 day in cyclosporine group with median time to onset of four days post transplantation. They did not find any association between CsA trough levels and occurrence hypertension, although it should be noted that trough levels may not be the optimal marker for prediction of CsA adverse effects. (28) Early and sometimes transient increase of blood pressure that could be irrelevant to cyclosporine exposure extent, again will endorse the requirement for frequent monitoring especially at initial post transplantation phase as we implemented.

Well-organized monitoring schedules could be a lifesaving and advantageous measure in management and modification of therapeutic process in quality of care for HSCT patients according to our findings. Prediction or at least early discovery of CsA adverse reactions or toxicities using experiments like this study will positively help the on-time decision making for implementation of supportive or therapeutic interventions. As mentioned before, increased post-discharge occurrence of CsA adverse effects parallel to elevated blood levels despite dose reductions, was a common observation. These complications could be the result of pharmacokinetic alterations during the time. (29) Considering our center protocol of weekly follow ups, this fact would suggest requirement for a more precise and frequent monitoring schedule with increased clinic visits or even telecommunications after patient discharge.

Considering clinical evaluations, acute GVHD occurred in significant number of our patients as seen in other studies. (30) While cyclosporine is utilized as the main part of GVHD prophylaxis in allogeneic HSCT regarding long term experience with it, evidence based support and financial concerns, any effort to optimize its application would be highly valuable. (31) Crucially health care system professionals should be acquainted with the standard dosage and administration approaches of this vital agent. Patient education and interviews about importance and sensitivity of this therapy is also vital for maintaining an appropriate therapeutic process. In addition providing efficient administration devices would be so influential in the pathway of achieving an optimal outcome. These results revealed facts about unawareness or negligence of health care staff for predefined CsA administration protocols and consequently probable imposition of harm to patients. It could be stated that developing more straight-forward, updating, evidence-based and globalized practice guidelines both for cyclosporine administration and monitoring will be of high value with the assistance of such trials in HSCT setting.
